# The theoretical and practical determination of clinical cut-offs for the British Sign Language versions of PHQ-9 and GAD-7

**DOI:** 10.1186/s12888-016-1078-0

**Published:** 2016-11-03

**Authors:** Rachel A. Belk, Mark Pilling, Katherine D. Rogers, Karina Lovell, Alys Young

**Affiliations:** 1 Social Research with Deaf People Group, Division of Nursing, Midwifery and Social Work, School of Health Sciences, University of Manchester, Manchester Academic Health Science Centre, Jean MacFarlane Building, Oxford Road, Manchester, M13 9PL UK; 2 Division of Nursing, Midwifery and Social Work, School of Health Sciences, University of Manchester, Manchester Academic Health Science Centre, Jean MacFarlane Building, Oxford Road, Manchester, M13 9PL UK

**Keywords:** British Sign Language, Improving Access to Psychological Therapies, IAPT, BSL, PHQ-9, GAD-7

## Abstract

**Background:**

The PHQ-9 and the GAD-7 assess depression and anxiety respectively. There are standardised, reliability-tested versions in BSL (British Sign Language) that are used with Deaf users of the IAPT service. The aim of this study is to determine their appropriate clinical cut-offs when used with Deaf people who sign and to examine the operating characteristics for PHQ-9 BSL and GAD-7 BSL with a clinical Deaf population.

**Methods:**

Two datasets were compared: (i) dataset (*n* = 502) from a specialist IAPT service for Deaf people; and (ii) dataset (*n* = 85) from our existing study of Deaf people who self-reported having no mental health difficulties. Parameter estimates, with the precision of AUC value, sensitivity, specificity, positive predicted value (ppv) and negative predicted value (npv), were carried out to provide the details of the clinical cut-offs. Three statistical choices were included: Maximising (Youden: maximising sensitivity + specificity), Equalising (Sensitivity = Specificity) and Prioritising treatment (False Negative twice as bad as False Positive). Standard measures (as defined by IAPT) were applied to examine caseness, recovery, reliable change and reliable recovery for the first dataset.

**Results:**

The clinical cut-offs for PHQ-9 BSL and GAD-7 BSL are 8 and 6 respectively. This compares with the original English version cut-offs in the hearing population of 10 and 8 respectively. The three different statistical choices for calculating clinical cut-offs all showed a lower clinical cut-off for the Deaf population with respect to the PHQ-9 BSL and GAD-7 BSL with the exception of the Maximising criteria when used with the PHQ-9 BSL. Applying the new clinical cut-offs, the percentage of Deaf BSL IAPT service users showing reliable recovery is 54.0 % compared to 63.7 % using the cut-off scores used for English speaking hearing people. These compare favourably with national IAPT data for the general population.

**Conclusions:**

The correct clinical cut-offs for the PHQ-9 BSL and GAD-7 BSL enable meaningful measures of clinical effectiveness and facilitate appropriate access to treatment when required.

## Background

The PHQ-9 [1] and the GAD- 7 [2] are two of the standard instruments mandated for use within the IAPT (Improving Access to Psychological Therapies) national (England) NHS (National Health Service) programme. IAPT is a large-scale initiative within the NHS (National Health Service) in England aimed at redressing long-standing imbalances between psychological therapy demand and supply. IAPT services deliver approved psychological interventions to address common mental health problems in primary care settings. The PHQ-9 [1] and the GAD- 7 [2] are used as screening and assessment tools, initially to indicate caseness (clinical threshold) as one indicator of eligibility for service. They are subsequently used at each session to assess progress leading to measurement of recovery and discharge ([3] p15). Patient and service data are also collected and analysed on a national basis ([3] p16).

Since December 2011, an adapted version of IAPT has been available, in a small number of geographical areas, to Deaf people who are users of British Sign Language (BSL) [4], henceforth BSL-IAPT. BSL is not a visual, transliterated version of spoken English [5]. It is an independent, fully grammatical visual-spatial language whose indigenous minority status was formally recognised by the UK government in 2003 [6] and its legal position strengthened in Scotland in 2015 [7]. Deaf people’s cultural/linguistic status is conventionally marked by the use of upper case ‘D’ (Deaf), rather than by lower case ‘d’ (deaf) which instead indicates being deaf without BSL use or its associated cultural identity [8]. IAPT for Deaf BSL users is particularly important because Deaf people are more than twice as likely to experience mental health problems than hearing people [9]. Deaf people’s access to health services is also much poorer than hearing people’s because of limited availability of information and treatment delivery in BSL [10, 11] and difficulties at point of access to services. Failure of services to address linguistic and cultural needs of Deaf people has been widely reported in the UK and in other countries [12].

BSL-IAPT uses the standard IAPT instruments, including PHQ-9 and GAD-7, but in their validated BSL translated form. These translations were carried out by authors 3, 4, 5 following strict protocols agreed with the originators of the instruments, and construct validity, internal reliability and test-retest reliability were found acceptable [13].^1^ The BSL versions of the standard instruments are delivered on screen, as video-recordings, because BSL is not a language with a written form. Although PHQ-9 BSL and GAD-7 BSL are now in current use, the BSL-IAPT service have used them in conjunction with the clinical cut-off scores adopted by IAPT ([3] p22): these scores were derived from studies that have only involved hearing populations using the English versions.

However, as is the case with any translated version of a standard instrument, the clinical cut-off that is in use for one cultural-linguistic population may not be appropriate for another; it cannot be assumed to have the same sensitivity and specificity as that for the population on which it was originally validated [14]. Field testing in the linguistic and cultural population in which the translated version is applied is required not only to measure operating characteristics of reliability and validity [15–17], but also to establish whether the clinical cut-off is the same or different. Such testing has been carried out for many translations of GAD-7 and PHQ-9 into languages other than English [18] and also with respect to English versions used with populations where there are cultural differences or particular distinguishing characteristics e.g. a group in another English-speaking country, one with a specific illness or one based in primary care [19–22].

The existence of a large dataset of Deaf patients who were referred to BSL-IAPT between December 2011 and February 2015 (*n* = 791), including the use of reliability-tested, standard BSL versions of the PHQ-9 and GAD-7, presented a unique opportunity to investigate the clinical cut-offs of the standard instruments in BSL when used with a primary care population for purposes of assessment and treatment. This paper reports the operating characteristics for PHQ-9 BSL and GAD-7 BSL and considers how different approaches to balancing sensitivity and specificity affect the selection of cut-offs. In this context sensitivity is the percentage of correctly identified unhealthy people, and specificity is the percentage of correctly identified healthy people. The proposed cut-offs are then retrospectively applied to the data from the Deaf BSL users seen by BSL-IAPT to consider how they would have affected eligibility to the service through the measure of ‘clinical caseness’ ([3] p39) relative to the English cut-offs. They are also used to calculate ‘recovery’ as defined by the IAPT national programme ([23] p3), ‘reliable improvement/reliable deterioration’ ([23] p4) and ‘reliable recovery’ ([23] p5, 25). A summary of the demographic characteristics of the datasets are reported so comparability between them can be judged.

## Methods

### Secondary data analysis

This study involves secondary data analysis of the two datasets: (i) BSL-IAPT clinical dataset; and (ii) dataset of self-reported well Deaf people derived from a previous study. The anonymised BSL-IAPT clinical dataset comprises all those referred from the inception of the service (December 2011) to February 2015 (*n* = 791) and is compared against the study inclusion and exclusion criteria to identify Dataset 1 (*n* = 502). As an IAPT service provider, BSL-IAPT is permitted to hold records of its clients’ characteristics, adherence and outcomes in accordance with the IAPT recommended data fields and client data security arrangements. Dataset 2 is a comparator group (*n* = 85) of Deaf people from our previous study of the validity and reliability of the PHQ-9 BSL and GAD-7 BSL [13]. These data were collected in 2011/2012 in a form that does not permit individual identification of participants, therefore available data on participant characteristics is restricted to those collected at the time and retrospective collection of further participant characteristics was not possible. The comparator group self-reported having no mental health difficulties in the 12 months prior to the study and none were a current patient under mental health services.

In calculating clinical cut-offs, some studies have evaluated PHQ-9 and GAD-7 against an alternative method of assessment for the same cohort e.g. a clinical interview such as SCID [24]. This was not an option because of the anonymous status of data to which we had access and the limits of our ethical approval. Therefore, our design compared the two datasets of self-defined ‘well’ Deaf people with ‘not well’ Deaf people, the latter defined as such by virtue of having been assessed by a MHP (Mental Health Practitioner) as eligible for therapy through IAPT. The analysis sought to define how well the two tests discriminated between the two groups (Dataset 1 and Dataset 2).

### Materials

The nine questions of the PHQ-9 score the nine DSM-IV criteria for depression by a frequency scale from 0 to 3 and the instrument is most commonly scored by the simple summing of the questions to give an overall total of 0 to 27. The originators of the instrument established a score of 10 as the clinical cut-off for moderate depression in the English version [1], measured against the ‘gold standard’ of an MHP interview. This score yielded a sensitivity of 88 %, a specificity of 88 % and a positive likelihood ratio of 7.1. GAD-7 is scored by a frequency scale from 0 to 3 for each item and is also most commonly totalled to give a score between 0 and 21. It was validated against other health measures and against an MHP interview. A clinical cut-off of 10 was identified against the MHP interview diagnosing generalised anxiety disorder (GAD) with a sensitivity of 89 % and a specificity of 82 % [2]. However, a later study [25] evaluated GAD-7 as a broader instrument to test for any anxiety disorder and determined an acceptable AUC of 0.86. From this AUC, a lower cut-off of 8 for any anxiety disorder was recommended, which gave a sensitivity of 77 %, a specificity of 82 % and a positive likelihood ratio of 4.4. This lower cut-off was the one adopted by IAPT to sit alongside that for the PHQ-9 ([3] p22).

We note that there are, to date, no published analyses of the operation of the clinical cut-off scores for both instruments with respect to the IAPT population in general. Patient characteristics in this population, in comparison with those on which the original cut-off scores for the English versions were originally derived, may indicate that a revision of the cut-off scores currently in use in IAPT services is required. However for the purposes of this study, we use the published IAPT-recommended cut-off scores.

### Ethics

Ethical permission was sought, and approved by, the Proportionate Review Sub-committee of NRES (National Research Ethics Service) Ref: 14/LO/2234 for transfer of the anonymised Dataset 1 to the research team at the University of Manchester for the purpose of secondary data analysis. The people whose data was held within Dataset 2 had given online consent specifically for secondary data analysis within other studies, in addition to consent for the study during which it was first collected. Ethical permission had been sought and approved at the time of its collection through NRES Ref: 11/YH/0180.

### Participants

**Fig. 1:**
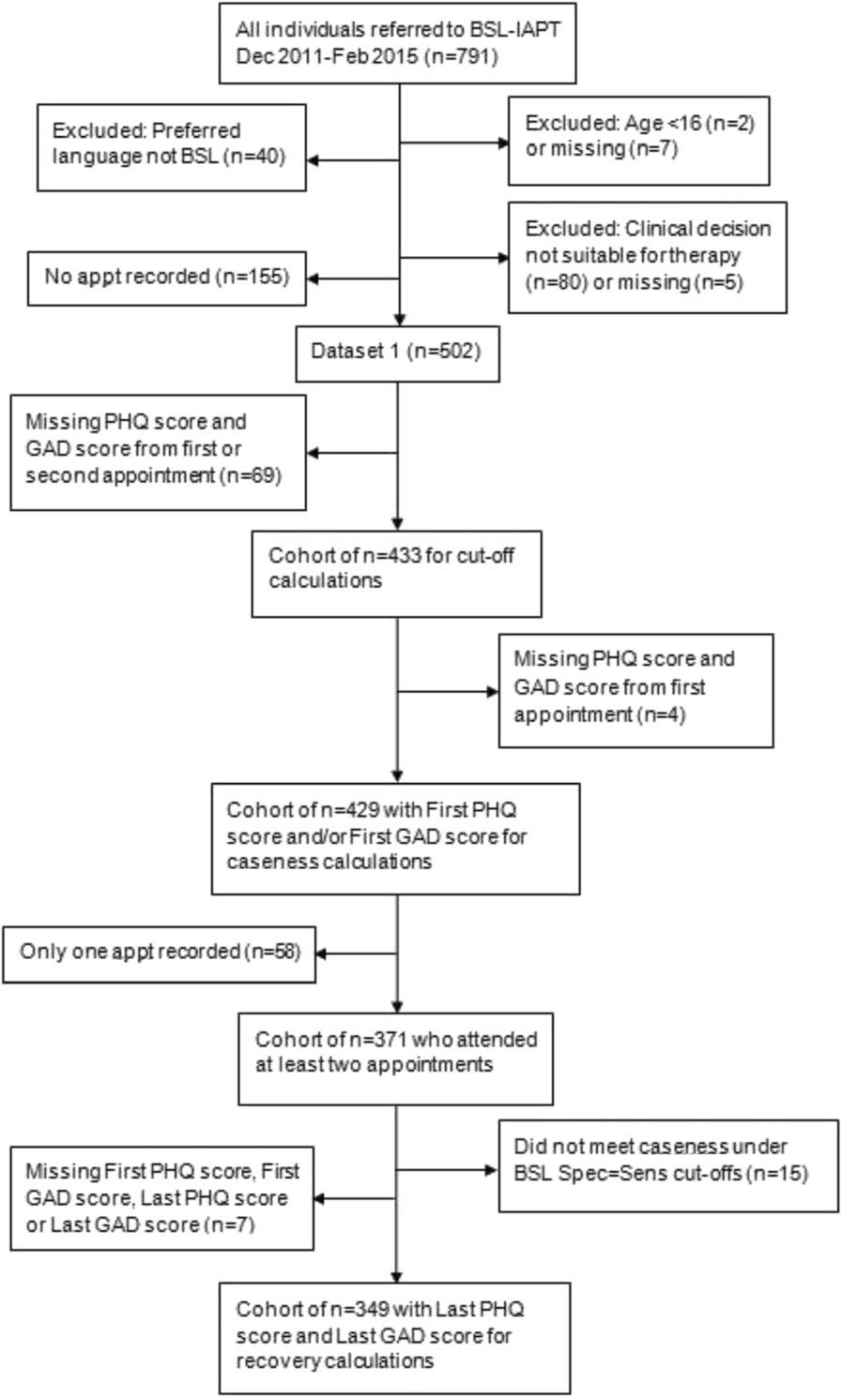
Consolidated Standards of Reporting Trials (CONSORT)-type diagram for the identification of Dataset 1

Figure 1 shows how the 791 people referred to BSL-IAPT were checked against the study inclusion and exclusion criteria to identify Dataset 1 (*n* = 502) and, within that, the cohorts used for each calculation. The inclusion criteria were that an individual was a Deaf sign language user, aged 16 years or over, had accessed BSL-IAPT services since December 2011, had received a step 2 or 3 service^2^ [26] and had attended a minimum of one therapist contact session. The 791 individuals referred to BSL-IAPT included 40 people who were not BSL users and were primarily spoken language users, two young people who were 14 and 15 years old, but had been assessed as being suitable to be seen by the adult service, those people who had been clinically judged not suitable for therapy through IAPT and those people who had had no appointment. These people were excluded. Of the latter group, the most common reason for no appointment was because the IFR (individual funding request) submitted for the person to attend a specialist service had been declined by the CCG (clinical commissioning group) or a decision was still pending. This reason is only applicable to referrals since Autumn 2014 as before this time, commissioning arrangements were different and the service had been commissioned as a whole rather than funding being sought for each individual referral [4].

### Analysis

The data were managed and analysed using IBM SPSS Statistics Version 22. The PHQ-9 and GAD-7 total scores were calculated using the guidelines in the IAPT Handbook ([3] p29), which allows the test still to be considered valid with up to two missing values. In such instances, one or two missing values can be replaced by a pro-rata value calculated by taking the mean of the 7 or 8 existing values. The total score is then calculated by 9 [mean value]. Preparatory sample size calculations were carried out based on Gilbody et al. [22], a study which observed a sensitivity of 91.7 % and specificity of 78.3 % for PHQ-9 as a screening tool for depression in 93 patients. We assumed a prevalence rate of 33 % for anxiety and/or depression in the Deaf population based on the well-cited Kvam et al. study [9] rather than more general estimates of mental health difficulties in the Deaf population. Following the same specificity and sensitivity as in the Gilbody et al. study [22], we estimated that a 90 % CI for an AUC to within +/−0.1 would require a sample size of at least 117 (39 depressed and 78 not-depressed patients). This calculation suggested that the numbers in the respective datasets would be sufficient.

Where new cut-offs have been determined for different populations, it is uncommon for authors to state clearly the statistical decisions based on clinical context that influenced the choice of cut-off. This includes studies where new cut-offs have been determined within different linguistic/cultural populations following translation.

The original papers determining PHQ-9 [1] and GAD-7 [2, 25] cut-offs did not specify exactly how they made a statistical choice between, for example, Maximising (Youden index) [27] or by Equalising sensitivity and specificity when they were choosing their cut-off. Kroenke et al. however do state that ‘at a GAD-7 cut-point of 8 or greater, sensitivity and specificity approached or exceeded 0.75 for all disorders and the positive likelihood ratio exceeded 3.0. The likelihood ratio is similar to that of most measures used to screen for depression in primary care.’ ([25] p321). It seems likely that, in their later review [28], they also used a cost function equalising sensitivity and specificity, though they do not state this explicitly.

For both PHQ-9 BSL and GAD-7 BSL, an AUC value with 95 % CI based on distributional theory was calculated. Different misclassification cost functions (e.g. Maximising, Equalising sensitivity and specificity) were then used to calculate cut-offs and measure sensitivity, specificity, error rate and positive likelihood ratio. Considering the discussions about cost function by Kroenke et al. [28] and Löwe et al. [29], we also calculated a cut-off which considered false negatives to be twice as bad as false positives (FN:FP = ~1:2).

Bootstrapping of the sample was used to estimate variability (i.e. 95 % CI) for cut-off values. Although the results for the different decisions are presented to show the variation in psychometric properties when different cut-offs are used, the cut-off proposed for future use is that which matches the conditions used by the originators of the tool i.e. Sensitivity = Specificity [28]. The bootstrapped 95 % CI for the new BSL cut-off was compared with the English cut-off for each test and a p value was calculated to see if there was a statistically significant difference between the clinical cut-off values.

The standard measures defined by IAPT were used in the analysis. ‘Caseness’ ([3] p39) pertains to entry into the service: an individual is defined as having reached caseness if they have a score equal to or higher than the cut-off on PHQ-9 and/or GAD-7 at assessment. The second IAPT-specific measure is ‘recovery’ ([23] p3): this is said to have been reached when a client’s PHQ-9 and GAD-7 scores both fall below the clinical cut-off and they were at ‘caseness’ at intake. Gyani et al. [30], in their detailed analysis of client data from the first year of IAPT operation, highlighted that ‘this measure does not take into account whether the observed change is greater than the measurement error of the scales’ ([30] p599). Additionally, a small improvement taking an individual from just above to just below the clinical cut-off is classified as recovery, whereas an individual who started with a high score on one or both instruments and has greatly improved, but did not fall below cut-off, is not counted. The additional use of a formula to calculate a reliable change index (RCI) [31], equivalent to a score change of at least twice the standard error, was therefore proposed by Gyani et al. [ibid]. The RCI enables the quantification of ‘reliable improvement’ and ‘reliable deterioration’ i.e. a score change larger than the RCI signals a clinically significant change. This measure, when combined with ‘recovery’, enables the identification of those individuals who have ‘reliably recovered’ i.e. shown both ‘recovery’ and ‘reliable improvement’. IAPT have recently moved to adopt the use of ‘reliable recovery’ alongside ‘recovery’ [23, 32]. Following this lead, the reliable change indices (RCI) for PHQ-9 BSL and GAD-7 BSL were calculated using Jacobson and Truax’s criteria formula [31]. The measure of reliability used in the calculation was the measure of internal reliability, Cronbach’s alpha: a choice supported by Evans et al. [33] and previously calculated by authors 3, 4, 5 [13].

The newly identified cut-offs and reliable change indices for PHQ-9 BSL and GAD-7 BSL were then retrospectively applied to Dataset 1 to calculate how many of the clients reached caseness, recovery and reliable recovery and how many showed reliable improvement or reliable deterioration. Reliable improvement is defined as a fall in score for one instrument greater than the RCI, whilst the score for the other instrument either also reliably improves or does not show reliable change. Reliable deterioration is the opposite; a rise in score for one instrument whilst the other instrument also shows reliable deterioration or no reliable change. Any other combination of score changes (e.g. one instrument shows reliable change, but the other shows reliable deterioration, or both show no reliable change) is labelled as no reliable change. Additional analysis of Dataset 1 allowed characterisation of the cohort in terms of demographics and origin of referral.

## Results

### Population characteristics

Datasets 1 and 2 were compared in respect of the available demographic descriptors to judge whether the groups were comparable (Table 1). Gender, age and ethnicity were available for both datasets and showed a similar male/female split, mean age (Dataset 2 was slightly skewed towards younger age brackets) and the proportion of respondents/clients who indicated that they were of White-British ethnicity. The question on disability to the participants in Dataset 2 did not exclude being deaf. Dataset 2 contained a higher proportion with a declared disability compared to Dataset 1. In the latter groups, type of disability was broken down so being deaf could be excluded and it was variable whether individuals indicated being deaf as a disability: this is likely to be the same for Dataset 2, although this cannot be confirmed from the available data.

**Table 1:** Description of Datasets 1 and 2 with respect to demographic characteristics

Demographic	Dataset 1 (*n* = 502)		Dataset 2 (*n* = 85)	
	Number/Valid number	%	Number/Valid number	%
Female gender	303/502	60.4	49/84	57.6
Age range	16–80/502		22–68/83	
Mean age	42 (13.2 SD)		40	
Ethnicity White-British	358/425	84.2	74/83	89.2
Religious belief Christian	140/215	65.1		
Sexual orientation Heterosexual	266/322	82.6		
Relationship: married/partner	151/376	40.2		
Relationship: single	153/376	40.7		
Relationship: divorced/widowed	72/376	19.1		
National Identity English	143/149	96.0		
Declared disability	45/502	9.0^a^	28/83	33.7^b^
Has long-term health condition	83/374	22.2		
Prescribed psychotropic medication	175/435	40.2		
Receiving sick pay	16/434	3.7		
In paid employment	110/433	25.4		
Previously accessed Standard IAPT	219/502	43.6		
Provisional diagnosis depression	120/414	29.0		
Provisional diagnosis anxiety	49/414	11.8		
Provisional diagnosis mixed anxiety and depression	208/414	50.2		
Provisional diagnosis other	37/414	8.9		
North West Region	323/502	64.3		
Primary care referral	192/502	38.2		
Self-referral	205/502	40.8		
Other referral source	105/502	20.9		

^a^Question excluded being deaf, ^b^Question did not exclude being deaf

### Establishing clinical cut-offs and reliable change indices

Table 2 shows the numbers within each dataset that were valid for calculating the cut-offs.

**Table 2:** Valid numbers of participants for calculating clinical cut-offs for PHQ-9 BSL and GAD-7 BSL

	Dataset 1 (Data from BSL-IAPT Deaf clients 2011–2015) *n* = 502		Dataset 2 (Data from self-reported healthy Deaf participants from Rogers et al. [13]) *n* = 85	
	Valid number of participants	Mean instrument score	Valid number of participants	Mean instrument score
PHQ-9 BSL Score	433	14.58 (SD = 5.99)	85	3.62 (SD = 3.29)
GAD-7 BSL Score	432	12.50 (SD = 4.98)	84	2.13 (SD = 2.48)

**Fig. 2:**
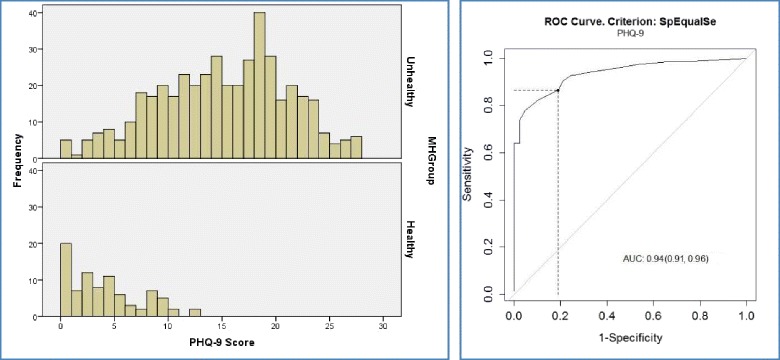
Distribution of PHQ-9 BSL scores for the two groups; ROC curve for PHQ-9 BSL

**Fig. 3:**
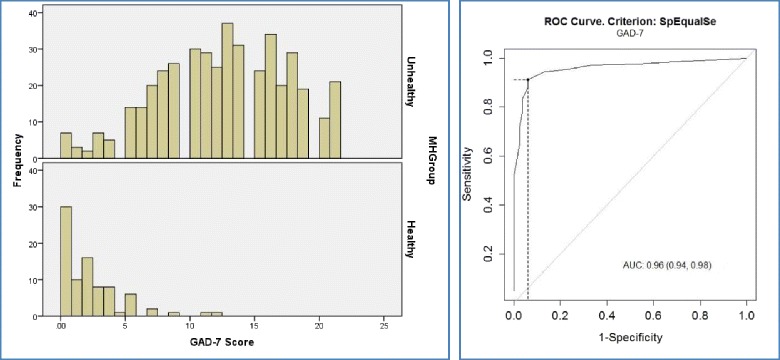
Distribution of GAD-7 BSL scores for the two groups; ROC curve for GAD-7 BSL

Figure 2 shows the distribution of PHQ-9 BSL scores for the two datasets and the ROC analysis. The AUC for PHQ-9 BSL was 0.94 with a 95 % CI of 0.91–0.96. Figure 3 shows the equivalent figures for GAD-7 BSL. The GAD-7 BSL tool had an AUC of 0.96 with a 95 % CI of 0.94–0.98. Both tools therefore show excellent discrimination.

Table 3 shows that, for the BSL versions, the sensitivity & specificity are high for the cut-offs corresponding to both the Maximising and Equalising functions. The overall error rates of both are fairly low, but when a cut-off that equalises sensitivity and specificity is used for PHQ-9 BSL, the higher sensitivity and lower specificity is a better balance between type I and type II errors, giving a lower overall error rate. The LR + =sens/(1-spec) criteria (i.e. that LR+ > 3) used by Kroenke et al. [25] in deciding the cut-off for GAD-7 was also passed by the BSL cut-offs. Kroenke et al. [ibid] also required that both sensitivity & specificity > =0.75, which the BSL cut-offs satisfy. The exception to this, of course, is the cut-offs for FN:FP = ~1:2, where the sensitivity is taken to be far more important than the specificity.

**Table 3:** PHQ-9 and GAD-7 cut-offs compared with PHQ-9 BSL and GAD-7 BSL cut-offs, indicating different statistical choices

English version (Cut-offs calculated on hearing population)			
Cut-off choice [95 % bootstrap CI]		PHQ-9	GAD-7
Equalising	sens = spec	10	8
	sens, spec	88 %, 88 % [28]	77 %, 82 % [28]
BSL version (Cut-offs calculated on Deaf population)			
Cut-off choice		PHQ-9 BSL	GAD-7 BSL
Maximising	Maximise sens + spec	10 [8.1, 13.2]	6 [5.1, 7.2]
	sens, spec	78 %, 95 %	91 %, 94 %
	Error	19.3 %	8.3 %
	LR+	16.5	15.3
Equalising	sens = spec	8 [6.5, 8.7]	6 [5.1, 7.2]
	sens, spec	86 %, 81 %	91 %, 94 %
	Error	14.5 %	8.3 %
	LR+	4.6	15.3
Prioritising treatment FN:FP = ~1:2	Cost function: false negative judged twice as bad as false positive	4 [2.1, 7.2]	3 [0.0, 3.9]
	sens, spec	96 %, 55 %	97 %, 67 %
	Error	10.8 %	7.8 %
	LR+	2.1	2.9

It was decided to match the choice made by the originators of the English instruments and recommend the cut-offs corresponding to sensitivity = specificity, thus allowing easier comparisons between users of the different language versions. This gives a PHQ-9 BSL clinical cut-off of 8 (in comparison to 10 for the original English version) and, for the GAD-7 BSL, a clinical cut-off of 6 (in comparison to 8 for the original English version). T-tests examined whether the English PHQ-9 and GAD-7 cut-offs (Equalising) are the same as PHQ-9 BSL and GAD-7 BSL (Equalising), based on 1000 bootstrap replicates to gain a 95 % CI for the cut-offs. These tests gave strong evidence (*p* = 0.0003, *p* = 0.0002 respectively) against the hypothesis that they are equal [34]. The conclusion was that the new cut-offs for PHQ-9 BSL and GAD-7 BSL are significantly different from the English cut-offs.

Table 4 shows the reliable change indices (RCI) calculated for PHQ-9 BSL and GAD-7 BSL. BSL values for the reliable change index were shown to be slightly higher than the values for the English version used with the hearing population.

**Table 4:** Reliable change indices for PHQ-9 and GAD-7 compared with PHQ-9 BSL and GAD-7 BSL

	English version (hearing population)		BSL version (Deaf population)	
	PHQ-9	GAD-7	PHQ-9 BSL	GAD-7 BSL
Cronbach’s alpha	0.89 ([1] p608)	0.92 ([2] p1094)	0.81 ([13] p115)	0.88 ([13] p116)
Standard deviation of pre-therapy scores^a^	N/A	N/A	5.52	4.49
Standard error of difference^b^	N/A	N/A	3.40	2.20
Reliable change index	5.20 ([30] p599)	3.53 ([30] p599)	6.66^b^	4.31^b^

^a^ *n* = 411 from those reaching caseness using the Equalising cut-off – see Table 5

^b^following Jacobson and Truax [32 p14]

### Caseness, recovery, reliable change and reliable recovery

Of the 502 patients in Dataset 1, 429 have a first PHQ-9 BSL score and/or a first GAD-7 BSL score. This would have been used by the service for establishing caseness (see Fig. 1). Table 5 illustrates the application of the English cut-off scores to this cohort of 429, in comparison with the application of the BSL cut-off scores, dependent on which statistical decision is applied.

**Table 5:** Number of clients within Dataset 3 (*n* = 429) meeting or not meeting caseness under the cut-off scores

Cut-off used	Number of clients meeting caseness under this cut-off	Number of clients not meeting caseness under this cut-off	Percentage of clients meeting caseness under this cut-off
English: Equalising	392	37	91.4
BSL: Maximising	406	23	94.6
BSL: Equalising	411	18	95.8
BSL: Prioritising (FN:FP = ~1:2)	423	6	98.6

The lower cut-offs for the BSL instruments mean that a larger proportion of those referred would have reached caseness and therefore potential eligibility for therapy under the service.

‘Recovery’ can be calculated for those clients with at least two appointments and who were at caseness at the start of therapy (*n* = 349) (Table 6).

**Table 6:** Dataset 1 recovery rates after a minimum of two appointments and starting therapy at caseness (*n* = 349)

Cut-off used	Number of clients reaching recovery under this cut-off	Number of clients not reaching recovery under this cut-off	Percentage of clients reaching recovery under this cut-off
English: Equalising	187	162	53.6
BSL: Maximising	157	192	45.0
BSL: Equalising	150	199	43.0
BSL: Prioritising	70	279	20.1

The lower cut-offs for the BSL instruments mean that the apparent recovery rate drops compared to when the English cut-off is applied to Dataset 1. However, recovery rates for the BSL-IAPT service are still comparable to the range for IAPT services nationally [30, 35], even when the lower BSL cut-offs are used. The cohort used in Table 6 includes clients who, for example, dropped out before the end of therapy, who were referred on to other services partway through therapy or those who were still in therapy at the time of data collection. If the cohort is narrowed to only those who have completed therapy (Table 7), the proportion who reached recovery is much higher than in Table 6.

**Table 7:** Dataset 1 recovery rates after a minimum of two appointments, starting at caseness and completed therapy (*n* = 226)

Cut-off used	Number of clients reaching recovery under this cut-off	Number of clients not reaching recovery under this cut-off	Percentage of clients reaching recovery under this cut-off
English: Equalising	160	66	70.8
BSL: Maximising	137	89	60.6
BSL: Equalising	131	95	58.0
BSL: Prioritising	63	163	27.9

‘Reliable recovery’, combining ‘reliable change’ with ‘recovery’, was calculated for the 226 clients from Dataset 1 who had at least two appointments, who had reached caseness at the start of therapy and who had completed therapy (Table 8).

**Table 8:** Dataset 1 reliable change/reliable recovery rates after a minimum of two appointments, starting at caseness and completed therapy (*n* = 226)

Cut-off and RCI used		Clients showing recovery	Clients showing reliable improvement	Clients not showing reliable change	Clients showing reliable deterioration	Clients showing reliable recovery
English version (English RCI)						
Equalising	Number	160	177	41	8	144
	Percentage	70.8	78.3	18.1	3.5	63.7
BSL version (BSL RCI)						
Maximising	Number	137	173	47	6	127
	Percentage	60.6	76.5	20.8	2.7	56.2
Equalising	Number	131	As above	As above	As above	122
	Percentage	58.0				54.0
Prioritising	Number	63	As above	As above	As above	62
	Percentage	27.9				27.4

78.3 % of clients showed reliable improvement using the English reliable change index, compared to 76.5 % using the BSL reliable change index. This drop is due to the RCI being slightly higher for the BSL instruments: a function of the lower internal reliability as measured by Cronbach’s alpha. The need for a bigger change on the BSL instruments in order to register as a clinically significant change also affects the number who showed reliable deterioration: 3.5 % using the English RCI compared to 2.7 % using the BSL RCI.

As would be expected, the measure of ‘reliable recovery’ shows the same trend as the measure of ‘recovery’: that the lower cut-offs for the BSL instruments indicate that a lower percentage of clients have recovered under this measure.

## Discussion

### Operating characteristics of PHQ-9 BSL and GAD-7 BSL

There were a number of factors, statistical and practical, to consider in deciding which cut-offs to recommend. The existing literature was carefully reviewed with the aim of matching, where possible, the same statistical priorities chosen by the originators of the instruments, which would lead to the choice of cut-offs which equalise specificity and sensitivity. In addition, the comparison of the error rates between the Maximising and Equalising criteria with our data (Table 3) showed a lower overall error rate when using the latter cut-offs. The cut-offs that will therefore be proposed to IAPT for future use (alongside the BSL instruments) are a score > =8 for PHQ-9 BSL as equivalent to a clinically significant level of depression and a score > =6 for GAD-7 BSL as equivalent to a clinically significant level of an anxiety disorder.

The majority of studies which determine the cut-off score for an assessment have a larger healthy population dataset than the clinical population dataset [36]. With this study, however, we have a larger dataset from the Deaf clinical population than the sample of well Deaf people. As a consequence, we know more precisely the empirical distribution of the clinical Deaf population and therefore have more precise estimates of sensitivity than of specificity.

The reasons for the different cut-off scores in the Deaf population are unknown. We suggest that further research is needed to explore the potential reasons, but there are a number of hypotheses. It could be that the composition of the cohorts, in terms of their mental health, is different from the cohorts tested by the originators of the tools when calculating the original English cut-offs. There is much research recognising the potential impact of characteristics of the studied sample on the psychometric properties of the instruments e.g. a population with concurrent health problems or a population based within primary versus secondary care [28]. In contrast, it has not often been acknowledged in the literature that the actual construct being examined i.e. depression or anxiety, may vary within a particular language and/or cultural community. For example, during reliability testing of PHQ-9 BSL [13], two components rather than one were extracted. Previous studies in hearing populations had found, almost universally, one component for PHQ-9. Two possible reasons were put forward in discussion: that depression is culturally determined differently amongst the Deaf population and/or that certain parts of the instrument measured facets that may be answered differently by Deaf people for other reasons e.g. experiencing a lack of motivation to socialise and meet people may be not as a result of feeling depressed, but rather be a response to many Deaf people’s normal experiences of social contexts where most of the hearing people within them are unable to communicate in BSL ([13] p117). This hypothesis is lent support in a validation study of the BSL version of EQ-5D-5 L (health questionnaire) [37], where a small number of Deaf people were interviewed to find out how they understood key terms contained within EQ-5D-5 L BSL. This revealed that everyday experiences of communication barriers could affect the conceptualisation of key terms e.g. when asked about ‘mobility’ difficulties, a reply could be influenced by considerations of whether an individual was concerned about how easy or not it would be to communicate when buying a train ticket [ibid].

### Implications of new recommended clinical cut-offs

The provision of the BSL-IAPT specialist service was in response to the fact that Deaf people experience significantly poorer mental health than the hearing population, with studies suggesting that the prevalence of some common mental health problems is twice as high [9, 10, 38]. Furthermore, studies have demonstrated the inaccessibility of health services to Deaf people who use British Sign Language [10, 38–45]. This includes mental health services, and can result in late diagnoses and loss of benefit from early preventative interventions [12]. Deaf people are often users of mental health services only when a difficulty has escalated to the point where secondary/tertiary care intervention is required [10, 40, 41]. BSL-IAPT, where available, provides an accessible primary mental health care intervention. The service is delivered by qualified Deaf practitioners who use BSL during therapy. This ensures a linguistically and culturally-matched mental health intervention without the requirement of an interpreter. Although the PHQ-9 BSL and GAD-7 BSL have been available for use by BSL-IAPT since inception, until now they have been used with the cut-offs that were determined for the original English versions, which had been determined using a hearing-only cohort. Our results show that the assumption that the same cut-offs should be applied to the Deaf population is flawed because it gives a worse outcome in terms of the clinical impact.

Applying the cut-offs that have been developed for the hearing population to the two datasets gives a higher overall error (i.e. a higher combined proportion of missed unwell individuals and well individuals wrongly assessed as unwell) compared to the proposed new cut-offs. Missed unwell individuals can result in further deterioration of their mental health, which in turn can be costly for individuals, wider society and the economy. The findings suggest that the lower cut-offs can improve the reliability and quality of IAPT services when delivered to Deaf people using the BSL instruments. This is not only the case for the individual monitoring of someone’s mental health during assessment and therapy, but creates a platform for future secondary data analysis of a large clinical cohort of Deaf people that will be trustworthy and more meaningful. Furthermore, the determination of lower clinical cut-offs means that Deaf BSL users should benefit from services at an earlier stage of mental health difficulties. The current cut-offs used for the hearing population run the risk of deteriorating mental health problems and this could prove costly in terms of both the financial implications and the impact on the individual who receives less timely interventions.

The IAPT service chose the cut-offs for the English version because of relatively high sensitivity at these levels. Papers working with these instruments and determining standard clinical cut-offs have broadly used a cost function that treats false positives and false negatives as being equally bad. However, Kroenke et al. ([28] p352) highlights that ‘one might choose a different cutpoint depending upon the population being assessed (community vs. primary care vs. mental health setting) and the purpose of the assessment (routine screening vs. evaluating suspected cases)’. Considering the known challenges for the Deaf population who use BSL to access services, there is a case to be made for using lower cut-off points. A cost function prioritising treatment was therefore calculated and could be used in primary care as a preliminary screening tool to judge which Deaf BSL users may benefit from an assessment by a specialist (or adapted standard) service that has the linguistic and cultural resources to carry out a fuller interview. The instruments are one element of the wider assessment.

### Proportion reaching caseness, recovery and reliable recovery

Retrospectively applying the new cut-offs PHQ-9 BSL > =8 and GAD-7 BSL > =6 to the cohort of Deaf BSL users referred to BSL-IAPT indicated that a greater proportion would have been at caseness and therefore eligible for therapy. This has implications for resourcing services. In addition, a smaller proportion are indicated to have recovered/reliably recovered using the new cut-offs, compared to the English cut-offs used in the national reporting, although it is of note that the levels of recovery still compare favourably with the national figures [35]. It was previously reported in the BSL Healthy Minds’ Evaluation Report, that for the first 20 months of operation [46] the recovery rate using the old clinical cut-offs was 75 %, compared to 70.8 % calculated in our larger study. However, calculations on our data using the new clinical cut-offs give 58 % reaching recovery and 54 % reaching reliable recovery, as defined by IAPT. These lower rates still reach the target set by the IAPT programme of at least 50 % reaching recovery [23]. As well as reflecting the quality of therapy provided by the service, there are likely to be other factors influencing recovery for this cohort.

With the additional barriers to accessing services, it can be hypothesised that clients may take longer to reach the service and therefore may have poorer mental health, and correspondingly higher scores on these instruments by the time they are seen. If this is the case, this may impact on the amount of improvement that is needed for a client’s score to reach the recovery cut-offs and, consequently, on the recovery rate. However, we would contend that showing reliable change rates gives a more balanced picture of progress through therapy. For example, individuals may take longer to recover if they have worse mental health to begin with, but may show faster or larger improvement scores even if recovery is not reached. Currently, it is not possible to make direct comparisons for the same timeframe as the nationally reported IAPT figures are for the predominantly hearing population and the newer measures of reliable change and reliable recovery were only adopted by IAPT in April 2015.

### Limitations

A limitation of our methodology was not having the resources to use a clinical interview. This would have provided a clinical ‘gold standard’ against which to measure the PHQ-9 BSL and GAD-7 BSL. Instead, in order to calculate the cut-offs, the methodology used discrimination between a group defined as having a mental health problem (Dataset 1) and a group who self-reported as not having a mental health problem. Whilst we consider that the choice of methodology was robust, it is different from that used by the originators of PHQ-9 and GAD-7. In order to validate further the clinical cut-offs for PHQ-9 BSL and GAD-7 BSL, the inclusion of a clinical interview would be required.

Dataset 2, the well group comparator, also had some limitations. The anonymous data were derived from a pre-existing study and it was not possible to retrospectively gain additional information about patient characteristics that would have enabled stronger judgement of comparability to be made between the two datasets. The dataset also relied on a self-definition of well, as judged by no current mental health difficulties and no use of a mental health service for the past 12 months. Although self-reporting could be seen as a limitation in that individuals may not have been truthful, there was additional evidence for better mental health of this group in that the mean scores of the BSL versions of the PHQ-9 and GAD-7 were significantly lower in comparison to the other group from the same study who self-reported that they had experienced mental health difficulties in the past 12 months [13]. Additionally, the sample in dataset 2 might be perceived as being healthier than the general population, which could in turn contribute to lower cut offs. However this is not the case; the mean score for depression, as measured by PHQ-9, for the sample of ‘healthy’ Deaf people from our previous study (REF: [13]) (mean score of 3.62) is higher than the commonly reported mean score of the control group in some studies of hearing populations (e.g. a mean score of 2.31 in the study of Reiner et al. [47]; and a mean score of 2.55 in the study of Hanwella, Ekanayake and de Silva [48]. Therefore healthy Deaf people in dataset 2 are not healthier than the general hearing population and this is unlikely to be the reason for lower clinical cut-offs.

We acknowledge there is a source of potential error in calculating caseness and reliable recovery for the Dataset 1 participants using the usual IAPT clinical cut-offs in comparison with the newly calculated BSL clinical cut-offs. To our knowledge, there are no published studies that have examined the operational characteristics of the clinical cut-offs for the GAD7 and PHQ 9 specifically with the general population of IAPT users and therefore some uncertainty remains as to whether the cut-offs derived from the original validation studies for the two instruments are appropriate for use within the IAPT service. Indeed, there continues to be recognition that, as discussed in the background section, clinical characteristics and statistical decisions both influence the selection of most appropriate cut-off [14]. We also acknowledge that there is greater uncertainty associated with the use of the clinical cut-offs for the two instruments as a screen for caseness in comparison with their use for diagnostic purposes [49–51]. It will be interesting in future studies to examine this issue also with respect to the Deaf population and the cut-offs now established for the instruments in BSL.

## Conclusions

The primary aim of this research was to explore the operating characteristics of the PHQ-9 BSL and GAD-7 BSL instruments within IAPT in order to improve reliability and quality when delivering therapies to Deaf people and using the BSL instruments. Appropriate clinical cut-offs for these instruments are now established for Deaf BSL users. Assessment of the clinical effectiveness of BSL-IAPT, both for clinical practice and to allow accurate comparison with mainstream IAPT services, can now be made. Comparison is important in the national (English) monitoring of IAPT services through the mandatory data that flows upwards to the HSCIC (Health and Social Care Information Centre) [35].

## Abbreviations

AUC: Area Under the Curve
BSL: British Sign Language
CCG: Clinical Commissioning Group
FN:FP = ~1:2: False Negatives considered twice as bad as False Positives
GAD-7: Generalized Anxiety Disorder 7-Items Scale
IAPT: Improving Access to Psychological Therapies
IFR: Individual Funding Request
PHQ-9: Patient Health Questionnaire
RCI: Reliable Change Index

## Footnotes

1 A third instrument, WSAS (the Work and Social Adjustment Scale) [52] was also translated alongside the PHQ-9 and GAD-7 [13] and is in use by BSL-IAPT. It is not addressed in this paper because the originators did not intend it to be used with a clinical cut-off as a standalone diagnostic and recovery tool. Rather, it is intended as ‘a self-report scale of functional impairment attributable to an identified problem’ [52].

2 Step 2 and Step 3 are part of the stepped care programme set out in the NICE guidelines and implemented within the IAPT programme [26]. Step 2 encompasses low-intensity interventions such as guided self-help and encouragement from a psychological well-being practitioner (PWP) and Step 3 is defined as high-intensity interventions such as weekly, one-to-one therapy sessions.
